# 873. Four Decades of Gender Representation Among First Authors in Burns Literature: A Systematic Analysis

**DOI:** 10.1093/jbcr/irag033.350

**Published:** 2026-04-06

**Authors:** Francesco M Egro, Sarah M Tepe, Kian Daneshi, Mare G Kaulakis, Christopher J Fedor

**Affiliations:** University of Pittsburgh Medical Center, Pittsburgh, PA; University of Pittsburgh Medical Center, Pittsburgh, PA; School of Population Health and Medicine, University of Sheffield, London, England; University of Pittsburgh Medical Center, Mercy Burn Center, Pittsburgh, PA; University of Pittsburgh Medical Center, Department of Plastic Surgery, Pittsburgh, PA

## Abstract

**Introduction:**

Gender diversity in medical research leadership remains a critical concern across medical specialties. This study presents the first comprehensive analysis of gender representation trends among first authors in burns and trauma literature over four decades.

**Methods:**

We conducted a systematic analysis of 18 275 articles from 12 major burns journals (1982-2025) using automated PubMed data mining. A novel multi-layer gender inference system combining gender-guesser library API, cross-reference resolution of initials, extended non-Western name databases, and statistical pattern recognition achieved 87.8% classification success (16 040 articles), with cross-reference resolution contributing an additional 22% coverage by converting author initials to full names. Statistical analysis included logistic regression for temporal trends, chi-square tests for journal differences, and Wilson confidence intervals for all proportions.

**Results:**

Of 16 040 successfully classified articles, 3694 (23.0%) had female first authors. Female representation increased significantly over time, from 17.3% in the 2000s to 38.0% in the 2020s. Logistic regression revealed a highly significant temporal trend (*p*<.001) with substantial increases in female representation across decades. The improvement was particularly pronounced from the 2000s (17.3% female) through the 2020s (38.0% female), representing more than doubling of female first authorship. Analysis demonstrated consistent upward trajectory with notable acceleration in recent years, reflecting broader efforts toward gender equity in academic medicine.

**Conclusions:**

This study documents significant progress in gender representation among burns literature first authors, with female participation more than doubling from 17.3% to 38.0% over the study period. However, overall representation remains below parity at 23.0%, indicating continued need for equity initiatives. The robust automated classification methodology, enhanced by cross-reference resolution achieving 87.8% coverage, provides a scalable framework for gender analysis across medical specialties. These findings support continued efforts to promote gender equity in burns research leadership while demonstrating the effectiveness of comprehensive name resolution techniques for bibliometric analysis.

**Applicability of Research to Practice:**

This project shows the great strides that the field has taken to create an environment in which both females and males can succeed clinically and academically.

**Funding for the study:**

N/A.

